# Risk of Permanent Dental Staining Following Exposure to Newer-Generation Tetracycline Derivatives in Children: A Systematic Review and Meta-Analysis

**DOI:** 10.3390/children13081029

**Published:** 2026-08-02

**Authors:** Carmen Machuca-Portillo, Cira Suárez-Marchena, Lucy Chandler-Gutiérrez, María José Barra-Soto, Carolina Caleza-Jiménez, Lydia López-del Valle, Juan J. Segura-Egea

**Affiliations:** 1 Pediatric Dentistry Division, Department of Stomatology, School of Dentistry, University of Sevilla, C/Avicena s/n, 41009 Sevilla, Spain; mmachuca@us.es (C.M.-P.); chandler@us.es (L.C.-G.); mbarra@us.es (M.J.B.-S.); 2 Building San Juan Medical Center, School of Dental Medicine, University of Puerto Rico, Medical Sciences Campus UPR, San Juan, PR 00936, USA; lydia.lopez1@upr.edu; 3 Endodontic Division, Department of Stomatology, School of Dentistry, University of Sevilla, C/Avicena s/n, 41009 Sevilla, Spain; segurajj@us.es

**Keywords:** doxycycline, minocycline, tigecycline, tooth discoloration, child, tetracycline derivatives

## Abstract

**Highlights:**

**What are the main findings?**

- Concerns about permanent tooth discoloration have traditionally limited the use of tetracycline antibiotics in children younger than 8 years.
- Newer-generation tetracycline derivatives, particularly doxycycline, differ pharmacologically from first-generation tetracyclines, but their dental safety remains uncertain.

**What are the implications of the main findings?**

- This systematic review and meta-analysis found no significant increase in the risk of permanent dental staining after short-course doxycycline use in children.
- The findings support reconsidering the routine avoidance of doxycycline in young children when clinically indicated, while further evidence is needed for minocycline and tigecycline.

**Abstract:**

**Background/Objectives**: Historical concerns regarding permanent dental staining have restricted the use of tetracycline antibiotics in children younger than 8 years. However, newer-generation tetracycline derivatives, particularly doxycycline, differ pharmacologically from first-generation tetracyclines and may present a lower risk of discoloration. This systematic review and meta-analysis evaluated the risk of dental staining associated with newer-generation tetracycline derivatives. **Methods**: It was conducted according to the PRISMA 2020 statement and registered in PROSPERO. PubMed/MEDLINE, Scopus, and Embase were searched. Methodological quality was assessed, and the certainty of evidence using the GRADE approach. A random-effects meta-analysis of comparative studies was performed using the risk difference (RD) as the summary effect measure. **Results**: Six observational studies fulfilled the eligibility criteria. Three studies reported no cases of dental staining following doxycycline exposure, whereas one study identified parent-reported discoloration in 2 of 18 evaluated children (11.1%). Dental staining was reported in 2 of 41 children (4.9%) treated with minocycline and in 2 of 12 children (16.7%) with tigecycline. Meta-analysis of the three comparative studies demonstrated no significant increase in the risk of dental staining among exposed children compared with controls (RD = 0.007; 95% CI −0.015 to 0.028; I^2^ = 0%). Methodological quality ranged from moderate to high. According to GRADE, the certainty of evidence was low for doxycycline/minocycline and very low for tigecycline. **Conclusions**: Current evidence, although limited, suggests that short-course doxycycline is associated with little or no clinically relevant risk of permanent dental staining in children younger than 8 years. In contrast, the available evidence for minocycline and tigecycline remains insufficient to establish their dental safety.

## 1. Introduction

Tetracycline (TC) antibiotics were first introduced in the 1950s and exhibit broad-spectrum activity against both Gram-positive and Gram-negative bacteria. They remain essential for the treatment of atypical pneumonia, melioidosis, brucellosis, Q fever, rickettsioses, and for malaria prophylaxis [1]. Shortly after their introduction, however, permanent dental discoloration and enamel hypoplasia were reported in exposed children, with the first description published by Shwachman et al. in 1956 [2]. These observations led the United States Food and Drug Administration (FDA) to issue a warning in 1963 recommending that tetracyclines should not be prescribed to children younger than 8 years of age or to pregnant or breastfeeding women unless the expected therapeutic benefits clearly outweighed the potential risks [3,4,5]. Additional adverse effects include increased hepatotoxicity during pregnancy [6] and transient inhibition of bone growth in premature infants [7].

The mechanism underlying TC-associated dental effects is attributed to the ability of these antibiotics to chelate calcium at physiological pH, forming tetracycline–calcium orthophosphate complexes that become incorporated into mineralizing dentin and enamel during tooth development [8]. Historically, reported rates of permanent dental staining among exposed infants and young children ranged from 23% to 92% [9], with the risk being strongly influenced by treatment dose, duration [10], and timing of administration during periods of active tooth mineralization [3].

Unlike first-generation tetracyclines, second- and third-generation derivatives—including doxycycline, minocycline, and tigecycline (a glycylcycline)—possess distinct pharmacokinetic and pharmacodynamic characteristics that may reduce their potential for dental deposition. These agents require lower doses, allow less frequent administration [11], and exhibit reduced calcium-binding affinity, which may explain their substantially lower propensity to cause permanent dental discoloration [12]. At the same time, increasing antimicrobial resistance and the need for effective therapeutic alternatives have expanded their use in pediatric infectious diseases, making their safety profile an increasingly important clinical issue.

Whether these newer tetracycline derivatives can be safely administered to children has become the subject of considerable debate [13]. Experimental studies have shown that doxycycline induces significantly less dental discoloration than tetracycline or oxytetracycline [14]. Likewise, previous narrative reviews concluded that the risk of doxycycline-associated permanent dental staining in children younger than 8 years is very low, generally below 1%, and found no convincing evidence of clinically relevant dental adverse effects following short-course exposure [12,15]. Despite these emerging data, current prescribing practices remain largely influenced by historical evidence derived from first-generation tetracyclines [15]. Nevertheless, the American Academy of Pediatrics, in the 2018 Red Book, concluded that doxycycline treatment for up to 21 days is safe for children of any age when clinically indicated [16,17,18]. Because doxycycline is now recommended as first-line therapy for several potentially life-threatening infections, including Rocky Mountain spotted fever, clarifying its true risk of permanent dental staining has become increasingly important for evidence-based clinical decision-making [18].

To date, no systematic review has quantitatively synthesized the available comparative clinical evidence regarding permanent dental staining associated with newer-generation tetracycline derivatives in children. Although this review encompasses newer-generation tetracycline derivatives as a group, the available clinical evidence is anticipated to be considerably more robust for doxycycline than for minocycline or tigecycline, owing to the greater use of doxycycline in pediatric clinical practice. Consequently, the strength of the conclusions is expected to differ among these agents.

Therefore, the aim of this systematic review and meta-analysis was to critically evaluate clinical studies published between 2000 and the present investigating the risk of permanent dental discoloration following oral administration of doxycycline, minocycline, or tigecycline in children younger than 8 years of age.

## 2. Materials and Methods

### 2.1. Protocol and Registration

This systematic review and meta-analysis was conducted in accordance with the Preferred Reporting Items for Systematic Reviews and Meta-Analyses (PRISMA) 2020 statement [19] (Supplementary File S1). The review protocol, including the research question, eligibility criteria, search strategy, outcomes of interest, and methods for study selection, data extraction, risk-of-bias assessment, and data synthesis, was established a priori to minimize the risk of reporting bias and ensure methodological transparency. The protocol was prospectively registered in the International Prospective Register of Systematic Reviews (PROSPERO) (registration number: CRD420261385872, accessed on 5 May 2026).

### 2.2. Review Question

The review question was developed using the PICO (Population, Intervention/Exposure, Comparison, Outcome) framework [20], which was considered appropriate to define the eligibility criteria and guide the literature search.

The PICO components were defined as follows:

Population (P): Children younger than 8 years of age.

Intervention/Exposure (I): Exposure to newer-generation tetracycline derivatives, including doxycycline, minocycline, and tigecycline.

Comparison (C): Children not exposed to tetracycline derivatives, when a comparison group was available.

Outcome (O): Permanent dental discoloration or other clinically detectable developmental dental alterations (e.g., enamel hypoplasia).

Based on this framework, the review question was formulated as follows: Among children younger than 8 years of age, does exposure to newer-generation tetracycline derivatives increase the risk of permanent dental staining compared with no exposure?

### 2.3. Eligibility Criteria

Eligibility criteria were established a priori according to the PICO framework [20].

Studies were considered eligible if they met all of the following criteria: (1) observational study design, including retrospective or prospective cohort studies, case–control studies, cross-sectional studies, retrospective follow-up studies, or case series; (2) children younger than 8 years of age who received doxycycline, minocycline, or tigecycline for the treatment of any infectious disease; (3) evaluation of permanent dental staining or other clinically detectable developmental dental alterations following antibiotic exposure; (4) publication in English from January 2000 onwards; and (5) a minimum sample size of 10 participants.

Studies were excluded if: (1) antibiotic exposure occurred after 8 years of age; (2) dental staining or developmental dental defects were not evaluated as study outcomes; (3) the investigated antibiotic was not doxycycline, minocycline, or tigecycline, or results for these agents could not be extracted separately; (4) the study evaluated only topical antibiotic administration; (5) the publication was an in vitro or animal study, review article, editorial, letter, conference abstract, or expert opinion; or (6) the sample size was fewer than 10 participants.

### 2.4. Search Strategy

A comprehensive and systematic literature search was performed independently by two reviewers (C.S.-M. and C.M.-P.) in the PubMed/MEDLINE, Web of Science, Scopus, and Embase databases. The search strategy combined controlled vocabulary (Medical Subject Headings [MeSH] and Emtree terms) with free-text keywords related to dental discoloration and newer-generation tetracycline derivatives.

The search strategy included combinations of the terms “tooth discoloration,” “dental staining,” “tetracycline,” “doxycycline,” “minocycline,” and “tigecycline.” Boolean operators (AND, OR) were used to combine search terms, and truncation was applied where appropriate to maximize search sensitivity. The search syntax was adapted to the indexing system and specific requirements of each database.

The final electronic search was conducted on 30 June 2026. No restrictions were applied regarding study design during the electronic search. Searches were limited to studies published from January 2000 onwards and to articles written in English. No restrictions were applied regarding geographic location or the type of infectious disease for which the antibiotic had been prescribed.

To identify any additional eligible studies, the reference lists of all included articles were manually screened. The complete search strategies for each database are presented in Table 1, ensuring the reproducibility of the search process.

### 2.5. Selection of Studies

Study selection was performed independently by three reviewers (C.S.-M., L.C.-G. and M.J.B.-S.) using a two-stage screening process. In the first stage, titles and abstracts retrieved from the electronic search were screened against the predefined eligibility criteria to identify potentially relevant studies. In the second stage, the full texts of all potentially eligible articles were independently assessed to determine their final eligibility for inclusion in the review.

**Figure 1:**
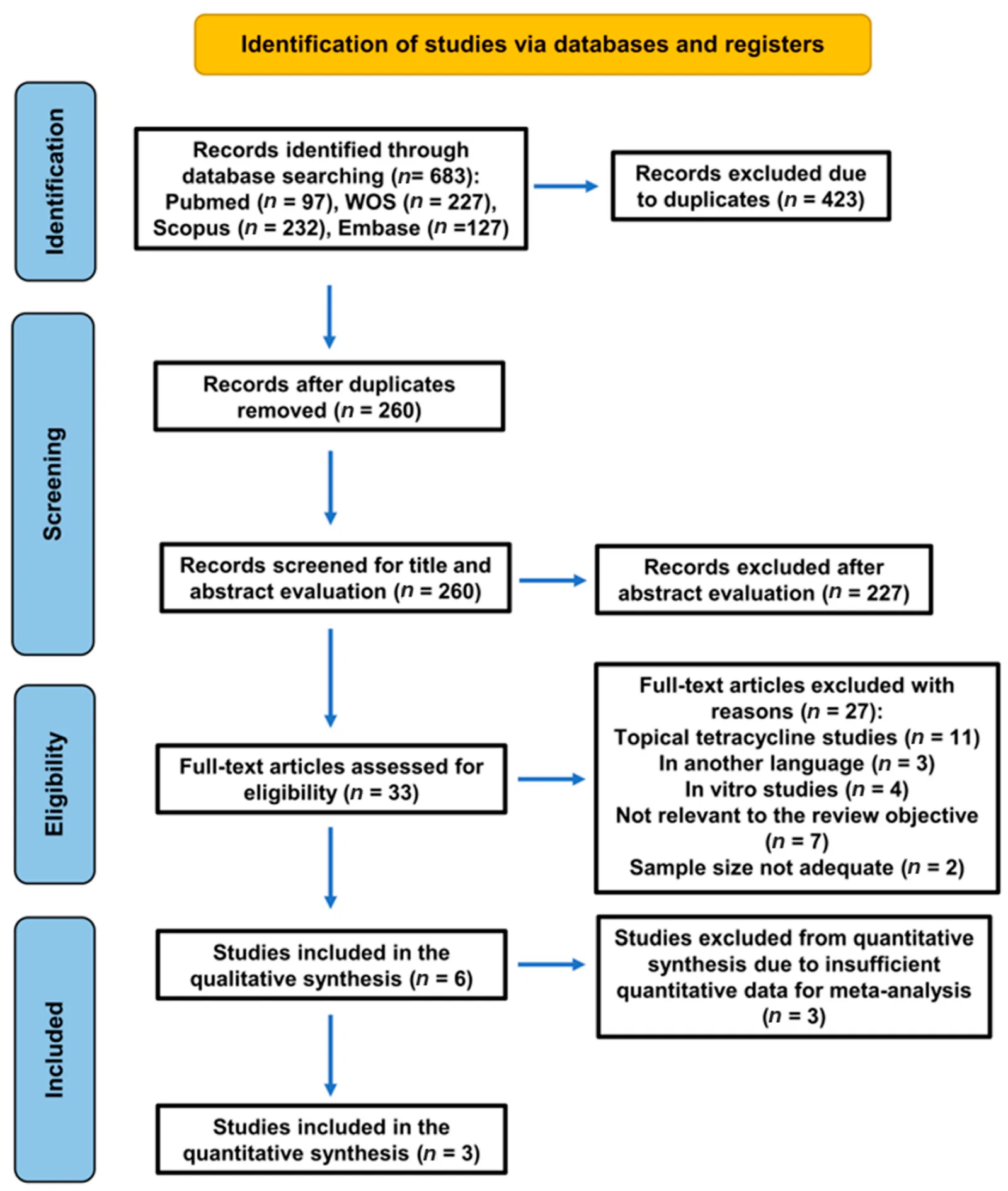
PRISMA flow diagram illustrating the study selection and screening process.

Disagreements at any stage of the selection process were resolved through discussion and consensus among the reviewers. The reasons for excluding studies after full-text assessment were documented and are presented in Table 2. The overall study selection process is summarized in the PRISMA 2020 [19] flow diagram (Figure 1).

### 2.6. Data Extraction

Data extraction was performed independently by two reviewers (C.M.-P. and L.L.-D.V.) using a standardized data extraction form specifically developed for this review. A third reviewer (J.J.S.-E.) cross-checked all extracted data to ensure accuracy, completeness, and consistency. Any discrepancies were resolved through discussion and consensus among the reviewers.

The following information was extracted from each included study: first author, year of publication, country, study design, sample size, participants’ mean age at antibiotic exposure, presence of a control group, type of tetracycline derivative evaluated, dosage regimen, treatment duration, age at dental follow-up, assessment of dental staining and other developmental dental alterations, main results, and authors’ conclusions. The extracted data were organized into standardized evidence tables for qualitative synthesis and quantitative analysis when appropriate.

### 2.7. Data Analysis

A qualitative synthesis was performed for all included studies. When at least two studies with comparable designs and outcomes were available, a quantitative synthesis (meta-analysis) was conducted. Because only three comparative studies were available, comparative studies evaluating newer-generation tetracycline derivatives were pooled irrespective of the specific compound.

The meta-analysis included studies that compared children exposed to newer-generation tetracycline derivatives with unexposed controls and reported the occurrence of permanent dental staining. The pooled effect size was expressed as the risk difference (RD) with corresponding 95% confidence intervals (95% CIs) using a random-effects model to account for potential between-study variability. Risk difference was selected because several included studies reported zero events in both the exposed and control groups.

Statistical heterogeneity was assessed using Cochran’s Q test and quantified with the I^2^ statistic, with values of approximately 25%, 50%, and 75% representing low, moderate, and high heterogeneity, respectively. A *p* value < 0.10 for Cochran’s Q test was considered indicative of statistically significant heterogeneity.

In addition to statistical heterogeneity, potential sources of clinical heterogeneity—including differences in the tetracycline derivative evaluated, treatment indication, dosage regimen, duration of therapy, follow-up period, and methods used to assess dental staining—were considered when interpreting the pooled estimates.

To evaluate the robustness of the pooled estimate, a leave-one-out sensitivity analysis was performed by sequentially excluding each study and recalculating the pooled effect size. Publication bias was not assessed because fewer than ten comparative studies were available, making funnel plot asymmetry tests unreliable.

All statistical analyses were performed using OpenMeta [Analyst] (Center for Evidence Synthesis in Health, Brown University, Providence, RI, USA). Statistical significance was established at *p* < 0.05.

To evaluate the robustness of the meta-analysis, a leave-one-out sensitivity analysis was conducted by sequentially excluding each comparative study and recalculating the pooled risk difference with the remaining studies. This analysis was performed to determine whether the overall estimate was disproportionately influenced by any individual study. Stability of the pooled effect across successive analyses was interpreted as evidence of the robustness of the meta-analytic findings.

### 2.8. Risk of Bias Assessment

The methodological quality of the included studies was assessed using study design-specific appraisal tools. The Joanna Briggs Institute (JBI) Critical Appraisal Checklist for Analytical Cross-Sectional Studies [48] was applied to the non-comparative observational studies, whereas the Newcastle–Ottawa Scale (NOS) [49] was used to assess the retrospective cohort studies.

Quality assessment was performed independently by two reviewers (C.M.-P. and J.J.S.-E.). Any disagreements were resolved through discussion and consensus.

The JBI checklist comprises eight methodological domains evaluating inclusion criteria, measurement of exposure and outcomes, identification and management of confounding factors, and the appropriateness of statistical analysis. Each item was rated as “Yes,” “No,” “Unclear,” or “Not applicable,” with higher numbers of positive responses indicating better methodological quality.

The Newcastle–Ottawa Scale evaluates cohort studies across three domains: selection of study groups, comparability of cohorts, and outcome assessment, with a maximum score of eight stars. Higher scores indicate lower risk of bias and greater methodological quality.

### 2.9. Analysis of GRADE Evidence Levels

The certainty of the evidence for the primary outcome (permanent dental staining following exposure to newer-generation tetracycline derivatives) was assessed using the Grading of Recommendations Assessment, Development and Evaluation (GRADE) approach [50]. The assessment considered the five standard GRADE domains: risk of bias, inconsistency, indirectness, imprecision, and publication bias, when assessable.

Three reviewers (C.M.-P., C.S.-M., and L.C.-G.) independently evaluated the certainty of the evidence. Any disagreements were resolved through discussion and consensus.

Because all included studies were observational, the certainty of evidence was initially rated as low, in accordance with GRADE methodology. The certainty rating was subsequently downgraded when serious concerns were identified in any of the assessed domains. Publication bias was not formally evaluated because the number of comparative studies available for quantitative synthesis was insufficient to support reliable assessment.

The overall certainty of evidence for each outcome was classified as high, moderate, low, or very low, according to the GRADE recommendations. A Summary of Findings table was prepared to summarize the certainty of the evidence for the primary outcomes.

## 3. Results

The study selection process is summarized in Figure 1. The electronic database search identified 683 records. After duplicate removal, 260 records remained for title and abstract screening. Following application of the predefined eligibility criteria, 33 full-text articles were assessed for eligibility.

Of these, 27 studies were excluded for the reasons detailed in Table 2. Consequently, six studies were included in the qualitative synthesis, of which three provided sufficient quantitative data to be included in the meta-analysis.

Ultimately, six studies fulfilled all eligibility criteria and were included in the qualitative synthesis [51,52,53,54,55,56]. Of these, four were retrospective cohort studies [51,52,53,54], whereas two were retrospective follow-up observational studies without a comparison cohort [55,56]. Three cohort studies provided sufficient comparative data to be included in the quantitative synthesis (meta-analysis). No prospective studies or randomized clinical trials meeting the eligibility criteria were identified.

The relatively small number of eligible studies highlights the limited amount of clinical evidence currently available regarding the dental safety of newer-generation tetracycline derivatives in young children.

### 3.1. Characteristics of the Included Studies

The main characteristics of the six studies included in the systematic review are summarized in Table 3. The studies were published between 2004 [51] and 2023 [56] and were conducted in Europe [51,54], Asia [52,55], and the United States [53,56]. Sample sizes ranged from 12 [55] to 58 children [53].

Four studies evaluated doxycycline [52,53,54,56], one investigated minocycline [51], and one assessed tigecycline [55]. Maintenance doses ranged from 2 mg/kg/day [53] to 5 mg/kg/day [54], although higher loading doses were administered in some studies [52,54]. Treatment duration varied from a mean of 7.3 days [53] to 21 days [51]. The mean age at dental follow-up ranged from 7.2 years [51] to 14.2 years [54].

Three studies included a non-exposed comparison group [51,52,53], whereas the remaining three were non-comparative retrospective observational studies [54,55,56]. Among the four studies evaluating doxycycline, three reported no cases of permanent dental staining [52,53,54], whereas Brown et al. [56] reported parent-reported dental discoloration in 2 of 18 evaluated children (11%). In contrast, dental staining was observed in 2 of 41 children (4.9%) treated with minocycline [51] and in 2 of 12 children (16.7%) exposed to tigecycline [55]. However, among the studies including an unexposed comparison group [51,52,53], no statistically significant differences in the occurrence of permanent dental staining were identified between exposed and control children (*p* > 0.05).

### 3.2. Dental Staining Outcomes

The dental staining outcomes reported by the included studies are summarized in Table 4. Overall, permanent dental staining was uncommon following exposure to newer-generation tetracycline derivatives. Among the four studies evaluating doxycycline [52,53,54,56], no clinically confirmed cases of permanent dental staining were reported in three studies [52,53,54], whereas Brown et al. [56] identified parent-reported dental discoloration in 2 of 18 children (11.1%) who completed dental follow-up. In contrast, the study evaluating minocycline reported clinically confirmed dental staining in 2 of 41 exposed children (4.9%) compared with 2 of 82 controls (2.4%), with no statistically significant between-group difference [51]. The only study investigating tigecycline reported dental staining in 2 of 12 evaluated children (16.7%) but did not include a comparison group [55].

Three studies included an unexposed control group [51,52,53]. None of these studies demonstrated a statistically significant increase in the occurrence of permanent dental staining among exposed children compared with controls. The remaining studies were descriptive and therefore did not permit comparative statistical analyses. Dental outcomes were assessed by clinical examination performed by trained dentists in all studies except Brown et al. [56], in which dental staining was reported by parents during telephone follow-up.

Although developmental dental alterations such as enamel hypoplasia were included as eligible outcomes, most studies focused primarily on clinically detectable permanent dental staining. Only Todd et al. [53] systematically assessed enamel hypoplasia in addition to tooth discoloration, whereas Cascio et al. [51] distinguished dental discoloration from other enamel defects.

### 3.3. Meta-Analysis

**Figure 2:**
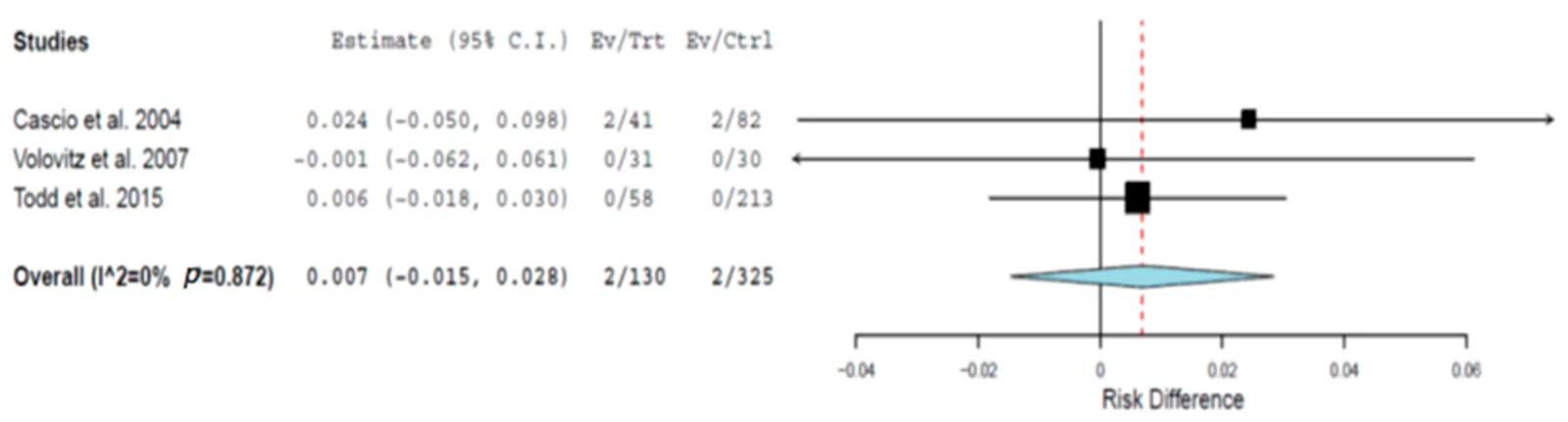
Forest plot of the meta-analysis comparing the risk of permanent dental staining between children exposed to tetracycline derivatives and unexposed controls [51,52,53].

A pooled meta-analysis was performed using the three studies that included a non-exposed control group (Figure 2). Because two studies reported zero dental staining events in both exposed and control groups, the effect size was expressed as the risk difference (RD) using a random-effects model. The pooled analysis showed no significant difference in the risk of dental staining between children exposed to tetracycline derivatives and unexposed controls (RD = 0.007; 95% CI = −0.015 to 0.028).

Overall, dental staining was reported in 2 of 130 exposed children (1.5%) and 2 of 325 controls (0.6%). Statistical heterogeneity was negligible (I^2^ = 0%, *p* = 0.872), indicating consistent findings across the included comparative studies. These findings should be interpreted cautiously because only three controlled studies were available, most of the evidence corresponded to doxycycline, and the pooled analysis combined different tetracycline derivatives.

### 3.4. Sensitivity Analysis

**Figure 3:**
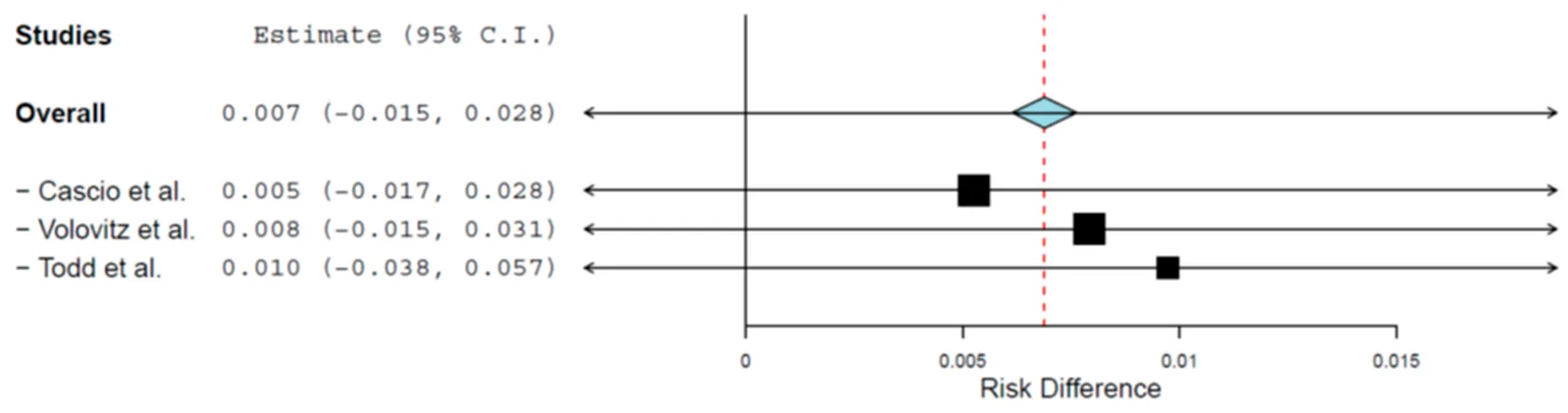
Leave-one-out sensitivity analysis of the meta-analysis evaluating the risk of permanent dental staining associated with tetracycline derivatives. Sequential exclusion of each study did not materially alter the pooled risk difference, indicating that no single study had a disproportionate influence on the overall estimate and confirming the robustness of the findings [51,52,53].

A leave-one-out sensitivity analysis was performed to evaluate the influence of individual studies on the pooled estimate (Figure 3). Sequential exclusion of each study produced only minimal changes in the pooled risk difference, which ranged from 0.005 (95% CI = −0.017 to 0.028) after excluding Cascio et al. [51] to 0.010 (95% CI = −0.038 to 0.057) after excluding Todd et al. [53]. Exclusion of Volovitz et al. [52] yielded a pooled risk difference of 0.008 (95% CI, −0.015 to 0.031). In all analyses, the confidence intervals included the null value, indicating that the overall findings were stable and were not driven by any individual study.

### 3.5. Risk of Bias Assessment

The methodological quality of the included studies was assessed using study design-specific appraisal tools. The Joanna Briggs Institute (JBI) Critical Appraisal Checklist for Analytical Cross-Sectional Studies was applied to the retrospective case series, whereas the Newcastle–Ottawa Scale (NOS) was used to assess the retrospective cohort studies (Table 5 and Table 6).

Among the two studies evaluated using the JBI checklist, Brown et al. [56] achieved a score of 7/8 because no strategies to control for confounding factors were reported. Zhu et al. [55] scored 6/8 owing to the absence of identification and statistical control of potential confounders. Nevertheless, both studies fulfilled most methodological quality criteria, indicating moderate-to-high methodological quality.

Among the four retrospective cohort studies assessed with the Newcastle–Ottawa Scale, Todd et al. [53] and Cascio et al. [51] achieved the maximum score (8/8), reflecting appropriate cohort selection, adequate follow-up, and objective outcome assessment. Volovitz et al. [52] and Pöyhönen et al. [54] each scored 6/8, mainly because of limited control for confounding factors and, in the latter study, the absence of a non-exposed comparison cohort. Overall, the cohort studies were judged to have moderate methodological quality, with NOS scores ranging from 6 to 8 out of 8.

For cohort studies, methodological quality was assessed using the Newcastle–Ottawa Scale (NOS). Of the four observational studies included, two studies achieved the maximum score of 8/8, and two scored 6/8. The highest-quality study was Todd et al. [53] and Cascio et al. [51], which used blinded dental examiners, objective spectrophotometric assessment of tooth colour, detailed exposure ascertainment, and adjustment for potential confounders. Lower scores were assigned to Volovitz et al. [52] and Pöyhönen et al. [54] because of limited control for confounding variables and, in the latter study, the absence of a non-exposed comparison cohort. Overall, the included studies were judged to be of moderate methodological quality. The main potential sources of bias were the retrospective design of most studies, limited control of confounding factors, absence of comparison groups in some studies, and relatively small sample sizes. The detailed assessment is presented in Table 6.

### 3.6. GRADE Assessment of the Certainty of Evidence

The certainty of the evidence for the main outcome (permanent dental staining following exposure to tetracycline derivatives during childhood) was assessed using the GRADE approach (Table 7). Observational studies start as low-certainty according to GRADE. The certainty of the evidence regarding the risk of permanent dental staining after short-course doxycycline or minocycline exposure in children younger than 8 years was rated as low. Although the included studies generally demonstrated moderate-to-high methodological quality and showed consistent findings, the certainty of the evidence was downgraded by one level because of serious imprecision resulting from the limited sample size and the rarity of dental staining events. For tigecycline exposure, the certainty of the evidence was rated as very low, owing to serious risk of bias and very serious imprecision related to the small sample size, substantial loss to follow-up, and the limited number of evaluated participants.

## 4. Discussion

Dental discoloration has historically represented the principal reason for restricting the use of tetracycline antibiotics during tooth development [2,3,4,5,56,57,58,59,60]. However, most of the evidence support ting this recommendation originates from studies performed several decades ago with first-generation tetracyclines [3,10,57,58,59,60], whereas considerably less information is available for newer tetracycline derivatives such as doxycycline, minocycline, and tigecycline [5,11,12,15]. This systematic review synthesized the available clinical evidence regarding the occurrence of permanent dental staining following exposure to these agents in children younger than 8 years of age [51,52,53,54,55,56]. In addition to the qualitative synthesis, a meta-analysis of controlled studies was performed, providing the first quantitative estimate of the comparative risk of dental staining associated with tetracycline derivatives during childhood.

### 4.1. Principal Findings

The present systematic review identified six observational studies evaluating the association between tetracycline derivatives and permanent dental staining in children [51,52,53,54,55,56]. Four studies investigated doxycycline [52,53,54,56], one evaluated minocycline [51], and one assessed tigecycline [55]. Overall, the available evidence suggests that short-course doxycycline administration during tooth development is associated with little or no clinically relevant risk of permanent dental staining. Three studies reported no cases of discoloration following doxycycline exposure [52,53,54], whereas a fourth study identified only two parent-reported cases without clinical confirmation [56]. Furthermore, the pooled analysis of the three controlled studies demonstrated no statistically significant increase in the risk of permanent dental staining among exposed children compared with unexposed controls, with negligible statistical heterogeneity, indicating highly consistent findings across comparative studies.

In contrast, the evidence for other tetracycline derivatives remains limited. The single study evaluating minocycline did not demonstrate a statistically significant increase in clinically confirmed dental discoloration compared with matched controls [51], although the available evidence is insufficient to establish its dental safety. Similarly, only one small retrospective study investigated tigecycline, reporting two cases of yellow discoloration among twelve examined children [55]. Given the limited sample size, absence of a control group, and substantial loss to follow-up, the certainty of evidence for tigecycline remains very low.

Taken together, these findings are consistent with the growing body of evidence indicating that doxycycline differs from older tetracyclines with respect to its potential to induce permanent dental staining [5,11,12,15]. The biological and clinical reasons underlying this more favorable safety profile are discussed below. Nevertheless, because the available evidence is derived exclusively from observational studies with relatively small sample sizes [51,52,53,54,55,56], the overall certainty of evidence remains low, and the results should be interpreted with appropriate caution.

Unlike previous narrative reviews, the present study provides the first quantitative synthesis of comparative evidence on dental staining associated with newer-generation tetracyclines and complements qualitative findings with risk-of-bias assessment, GRADE evaluation, and meta-analysis.

### 4.2. Doxycycline and the Risk of Permanent Dental Staining

Among the tetracycline derivatives evaluated, doxycycline was by far the most extensively investigated, accounting for four of the six included studies [52,53,54,56]. Overall, the available evidence consistently suggests that short-course doxycycline administration in children younger than 8 years is associated with a negligible risk of clinically relevant permanent dental staining. Three studies found no cases of dental discoloration following doxycycline exposure [52,53,54], while the only study reporting staining identified two parent-reported cases during telephone follow-up without clinical confirmation [56]. Moreover, the pooled analysis of the three comparative studies demonstrated no significant increase in the risk of dental staining compared with unexposed controls, further supporting the consistency of these findings.

The favorable safety profile of doxycycline is biologically plausible. Unlike first-generation tetracyclines, doxycycline exhibits lower affinity for calcium ions, resulting in reduced incorporation into mineralizing dental tissues [11,12,61]. This pharmacological characteristic probably explains why historical estimates of dental discoloration associated with older tetracyclines, which ranged from 23% to 92% [10,60,62], differ markedly from the findings observed in contemporary studies evaluating doxycycline [52,53,54,56]. Consequently, the long-standing contraindication applied to all tetracycline-class antibiotics may not accurately reflect the pharmacological properties of doxycycline. In addition to its lower calcium-binding affinity, doxycycline differs from first-generation tetracyclines in several pharmacokinetic characteristics that may further reduce its incorporation into developing dental tissues. Doxycycline is more lipophilic, exhibits higher protein binding, and is administered at lower therapeutic doses than older tetracyclines, resulting in lower concentrations of free drug available to chelate calcium during tooth mineralization. Furthermore, permanent dental staining occurs primarily during periods of active enamel and dentin mineralization, when tetracycline–calcium complexes become incorporated into the mineralizing matrix. Short therapeutic courses of doxycycline are therefore less likely to coincide with sufficient drug deposition to produce clinically detectable discoloration, particularly when compared with the prolonged or repeated exposure associated with first-generation tetracyclines.

The clinical settings represented in the included studies also strengthen the generalizability of these findings. Doxycycline was administered for a variety of indications, including atypical pneumonia [52], Rocky Mountain spotted fever [53], Lyme borreliosis [54,56], and other bacterial infections requiring short therapeutic courses. Importantly, the absence of clinically detectable staining was consistently observed despite differences in treatment duration, cumulative dose, and patient age at exposure [52,53,54]. Todd et al. [53] additionally demonstrated no increase in enamel hypoplasia or objectively measured tooth shade differences between exposed and unexposed children, while Pöyhönen et al. [54] reported no staining despite relatively high doses and treatment durations of up to 28 days.

These observations are consistent with previous narrative reviews and expert opinions suggesting that doxycycline differs substantially from older tetracyclines regarding its potential to induce permanent dental discoloration [5,12,15]. Similarly, earlier clinical reports by Lochary et al. [63] and Cale and McCarthy [64] described either no clinically significant discoloration or changes that were imperceptible to the naked eye after doxycycline exposure during childhood. Collectively, the available evidence supports current recommendations from the American Academy of Pediatrics, which no longer considers short courses of doxycycline to be contraindicated in children of any age when clinically indicated, particularly for potentially life-threatening infections such as Rocky Mountain spotted fever [18]. Nevertheless, despite the consistency of the available studies, the overall certainty of evidence remains low because all included investigations were observational and involved relatively small sample sizes.

### 4.3. Evidence for Minocycline and Tigecycline

In contrast to doxycycline, the available evidence regarding the dental safety of minocycline and tigecycline remains scarce. Only one study was identified for each antibiotic [51,55], precluding meaningful comparisons and preventing robust conclusions regarding their risk of permanent dental staining.

The study by Cascio et al. [51] evaluated the use of oral minocycline in combination with intravenous rifampicin for the treatment of childhood brucellosis. Although the authors reported no statistically significant increase in clinically confirmed dental discoloration compared with matched controls, the evidence should be interpreted cautiously. While minocycline possesses several pharmacological advantages over older tetracyclines, including improved oral absorption, greater lipophilicity, enhanced antimicrobial activity, and lower calcium-chelating capacity [65,66,67,68,69,70], these theoretical characteristics have not been adequately validated by clinical studies evaluating dental development. Consequently, despite the reassuring findings reported by Cascio et al. [51], there is currently insufficient evidence to conclude that minocycline shares the favorable dental safety profile observed with doxycycline.

Similarly, evidence regarding tigecycline is limited to a single retrospective follow-up study including only twelve evaluated children [25]. Tigecycline is a glycylcycline derivative with broad-spectrum activity against multidrug-resistant microorganisms [71] and is generally reserved as salvage therapy for severe pediatric infections when alternative treatments are unavailable or ineffective [72,73,74,75,76,77,78,79]. Zhu et al. [55] reported yellow discoloration in two of the twelve examined children following tigecycline exposure, corresponding to an incidence of 16.7%. However, the study was limited by the absence of a control group, substantial loss to follow-up, and a very small sample size. Consequently, although these findings suggest that tigecycline may retain the potential to induce permanent dental discoloration, the certainty of evidence is very low, and further well-designed studies are required before definitive conclusions can be drawn.

Overall, the available evidence indicates important differences in the amount and quality of evidence among newer tetracycline derivatives. Whereas doxycycline has now been evaluated in several independent clinical studies with largely consistent findings [52,53,54,56], the evidence supporting the dental safety of minocycline and tigecycline remains insufficient. Accordingly, the favorable conclusions reached for doxycycline should not be extrapolated to minocycline or tigecycline, as the currently available evidence for these agents is limited to isolated observational studies and remains insufficient to establish their dental safety.

### 4.4. Comparison with Previous Studies

The findings of the present systematic review are consistent with previous narrative reviews suggesting that doxycycline carries a substantially lower risk of permanent dental staining than first-generation tetracyclines [5,12,15]. Earlier reviews concluded that the available evidence did not support the historical contraindication of doxycycline in young children, particularly when short treatment courses were administered for serious bacterial infections. Likewise, Wormser et al. [5] questioned whether recommendations derived from older tetracyclines should continue to be extrapolated to doxycycline, given its distinct pharmacological characteristics and lower affinity for calcium.

However, the present review extends the existing literature in several important respects. First, it incorporates more recent clinical evidence, including studies published after the most frequently cited reviews [55,56]. Second, unlike previous narrative reviews, it systematically evaluated the methodological quality of the included studies using validated risk-of-bias tools and assessed the certainty of evidence using the GRADE framework. Third, it provides the first quantitative synthesis of comparative studies through meta-analysis, demonstrating no statistically significant increase in the risk of permanent dental staining among children exposed to newer tetracycline derivatives compared with unexposed controls.

Despite these advances, the conclusions of the present review remain appropriately cautious. Although the available evidence consistently supports the dental safety of short-course doxycycline, it remains based exclusively on observational studies and relatively small numbers of exposed children. Furthermore, the evidence for minocycline and tigecycline continues to be insufficient, highlighting that the favorable safety profile demonstrated for doxycycline should not automatically be extrapolated to other tetracycline derivatives until additional clinical evidence becomes available. This interpretation is also supported by the recently published systematic review and meta-analysis evaluating the dental safety of short-course doxycycline in children younger than 8 years, published by Rajan et al. [80]. Their findings are consistent with those of the present review, reporting a very low frequency of dental adverse events following short-course doxycycline use while emphasizing that the available evidence is based on a limited number of observational studies. The concordance between both reviews further supports the view that short-course doxycycline appears to be associated with a minimal risk of permanent dental staining when clinically indicated, although additional well-designed prospective studies are still needed to strengthen the certainty of the evidence.

### 4.5. Clinical Implications

The findings of this systematic review have important implications for pediatric clinical practice. Historically, the recommendation to avoid tetracycline antibiotics in children younger than 8 years has been extrapolated from evidence obtained with first-generation tetracyclines, particularly tetracycline itself, which exhibits a high affinity for calcium and a well-documented association with permanent dental staining [3,10,15,57,58,59,60,61,62]. However, the present review suggests that this historical concern should not necessarily be generalized to all newer tetracycline derivatives, particularly doxycycline.

The available evidence generally supports the favorable dental safety profile of short-course doxycycline when used for appropriate clinical indications. This conclusion is reinforced by the absence of an increased risk of permanent dental staining in the pooled analysis of controlled studies and is consistent with current recommendations from the American Academy of Pediatrics and other expert bodies, which recognize doxycycline as the treatment of choice for potentially life-threatening infections such as Rocky Mountain spotted fever regardless of patient age [18]. Similarly, doxycycline has become an important therapeutic option for Lyme disease, atypical pneumonia, and other infections in which its excellent antimicrobial activity, oral bioavailability, and tissue penetration offer clear clinical advantages [15,72,73].

From an antimicrobial stewardship perspective, unnecessary avoidance of doxycycline because of concerns derived from older tetracyclines may inadvertently lead to the use of broader-spectrum or less effective alternatives, prolonged hospitalization, or the need for intravenous therapy. Therefore, when doxycycline represents the recommended first-line treatment and no equally effective alternative exists, the available evidence suggests that concerns regarding permanent dental staining should not preclude its use in children younger than 8 years.

Nevertheless, these conclusions should not be extrapolated to other tetracycline derivatives. The evidence supporting the dental safety of minocycline and tigecycline remains limited and is based on isolated observational studies with small sample sizes [51,55]. Until more robust clinical evidence becomes available, their use in young children should continue to be guided by a careful assessment of the balance between expected therapeutic benefit and the potential risk of adverse effects on dental development.

### 4.6. Strengths and Limitations

This systematic review has several strengths. To our knowledge, it is the first systematic review and meta-analysis specifically evaluating the risk of permanent dental staining associated with newer-generation tetracycline derivatives in children younger than 8 years of age. The review was conducted according to the PRISMA 2020 guidelines, included a comprehensive search of multiple electronic databases, and applied predefined eligibility criteria. Study selection, data extraction, and quality assessment were performed independently by multiple reviewers, reducing the likelihood of selection and extraction bias. In addition, methodological quality was assessed using validated study design-specific tools, the certainty of evidence was evaluated using the GRADE approach, and a meta-analysis of controlled studies was performed to provide a quantitative estimate of the comparative risk of dental staining.

Nevertheless, several limitations should be acknowledged. First, the available evidence was limited to six observational studies, most of which included relatively small sample sizes and very few dental staining events. Second, only three studies included an unexposed comparison group, restricting the statistical power of the meta-analysis. Third, important clinical heterogeneity existed among studies regarding the tetracycline derivative evaluated, treatment indication, dosage, duration of therapy, follow-up period, and methods used to assess dental discoloration. Although statistical heterogeneity was negligible in the pooled analysis, these clinical differences should be considered when interpreting the results.

Additional sources of heterogeneity should also be considered. The included studies differed in the age at antibiotic exposure, cumulative dose, treatment duration, and interval between exposure and dental examination, all of which may influence the likelihood of detecting permanent dental alterations. Furthermore, although most studies assessed dental outcomes through clinical examination performed by dentists, the methods used for outcome assessment and the degree of examiner calibration were not uniformly reported. One study relied exclusively on parent-reported outcomes rather than clinical examination. These methodological differences may have contributed to variability in outcome ascertainment despite the absence of statistical heterogeneity in the pooled analysis.

Supplementary limitations arise from the methodological characteristics of the primary studies. Most investigations were retrospective, making them susceptible to incomplete clinical records and residual confounding. Furthermore, one study relied exclusively on parent-reported outcomes without clinical dental examination [56], whereas another experienced substantial loss to follow-up [55]. Finally, the evidence available for minocycline and tigecycline was limited to a single study for each antibiotic, precluding meaningful comparisons between tetracycline derivatives and preventing firm conclusions regarding their relative safety.

Taken together, these limitations explain why the certainty of evidence was rated as low for doxycycline and minocycline and very low for tigecycline despite the generally favorable methodological quality of the included studies. Consequently, although the available evidence is reassuring, particularly for doxycycline, the findings should be interpreted with appropriate caution until larger prospective studies become available. Moreover, because most studies focused exclusively on visually detectable permanent dental staining, the available evidence regarding other developmental dental defects, such as enamel hypoplasia, remains limited.

### 4.7. Future Research

Although the available evidence is generally reassuring, particularly for doxycycline, additional high-quality research is required to strengthen the current evidence base. Given the ethical constraints associated with randomized controlled trials in this field, future investigations should focus on well-designed prospective multicenter cohort studies with standardized protocols for antibiotic exposure, long-term follow-up, and comprehensive dental assessment. Larger sample sizes would improve the precision of risk estimates and allow meaningful subgroup analyses according to age at exposure, cumulative dose, treatment duration, and number of treatment courses.

Future studies should also adopt standardized and objective methods for assessing dental outcomes. Clinical examinations performed by calibrated examiners should be complemented, whenever possible, by objective color measurements, standardized photography, and radiographic evaluation to detect both visible discoloration and potential developmental alterations of enamel and dentin. Recording potential confounding factors, including oral hygiene practices, fluoride exposure, nutritional status, medical history, and concomitant medications, would further improve the validity of future observational studies.

Finally, additional research is particularly needed for minocycline and tigecycline, for which the current evidence is based on only one observational study each. Future investigations should determine whether the lower calcium-binding affinity of these newer tetracycline derivatives translates into a clinically meaningful reduction in dental adverse effects comparable to that observed with doxycycline. Such evidence would help refine current pediatric prescribing recommendations and support evidence-based decision-making when these antibiotics are required for the treatment of severe or drug-resistant infections.

## 5. Conclusions

Historical concerns regarding permanent dental staining have limited the use of tetracycline antibiotics in young children. This systematic review indicates that the available evidence, although limited, consistently suggests that short-course doxycycline appears to be associated with little or no clinically relevant risk of permanent dental staining. In contrast, evidence regarding minocycline and tigecycline remains insufficient to draw firm conclusions.

The pooled analysis of controlled studies found no significant increase in the risk of permanent dental staining among children exposed to tetracycline derivatives compared with unexposed controls. Although the overall certainty of evidence was low because of the observational nature of the available studies and the limited number of events, the current evidence supports consideration of short-course doxycycline, when clinically indicated, particularly in situations where it represents the recommended treatment and no equally effective alternative is available. Importantly, these conclusions should be interpreted in the context of the overall low certainty of the available evidence according to the GRADE assessment, reflecting the observational nature of the included studies and the limited number of events.

## Figures and Tables

**Table 1:** Literature search in databases.

Database	Exact Search String Used	No. of Articles	Date of Last Search
PubMed/ MEDLINE	(“minocycline” [MeSH Terms] OR “Tetracycline” [MeSH Terms] OR “Doxycycline” [MeSH Terms] OR “Tigecycline” [MeSH Terms] AND (“tooth discoloration” [MeSH Terms] OR “dental staining” [Title/Abstract])	97	From 2000 to 30 June 2026
Scopus	TITLE-ABS-KEY (“minocycline” OR “Tetracycline” OR “Doxycycline” OR “Tigecycline”) AND “antibiotics” AND (“tooth discoloration” OR “dental staining”)	232	From 2000 to 30 June 2026
Web of Science	(minocycline OR tetracycline OR doxycycline OR tigecycline) AND (“tooth discoloration” OR “tooth staining” OR “dental staining” OR “dental discoloration” OR “tooth pigmentation” OR “tooth colour” OR “tooth color”)	227	From 2000 to 30 June 2026
EMBASE	(‘minocycline’ OR ‘tetracycline’ OR ‘doxycycline’ OR ‘tigecycline’) AND ‘antibiotics’ AND (“tooth discoloration” OR ((‘tooth’/exp OR ‘tooth’) AND ‘discoloration’) OR “dental staining” OR (‘dental AND staining’))	127	From 2000 to 30 June 2026

**Table 2:** Excluded studies and their reasons for exclusion.

Reasons	Excluded Studies
Studies with topical use	AlSaeed et al., 2018 [21] Venkataraman et al., 2019 [22] Küçükekenci et al., 2019 [23] Porter et al., 2016 [24] Altun & Turkyilmaz, 2024 [25] Kirchhoff et al., 2015 [26] Nagata et al., 2014 [27] Tay et al., 2006 [28] Akcay et al., 2014 [29] Kim et al., 2010 [30] Mandras et al., 2013 [31]
In another language	Bremell & Trollfors, 2017 [32] Li et al., 2004 [33] Chen et al., 2003 [34]
In vitro studies	Bennett & Walsh, 2015 [35] Newman et al., 2025 [36] Chan et al., 2006 [37] Fukuta et al., 2001 [38]
Did not correspond with objective of review	Metz et al., 2017 [39] Tapananon et al., 2025 [40] Leonard et al., 2003 [41] Gimeno et al., 2008 [42] Deliperi et al., 2006 [43] Chen et al., 2005 [44] Matis et al., 2002 [45]
Sample size not adequate	Ayaslioglu et al., 2005 [46] Joshi et al., 2020 [47]

**Table 3:** Characteristics of the Included Studies.

Authors, Year, Country	Sample Size/Mean Age	Study Design	Control Group	AB	Dose Regimen	Treatment Duration	Follow-Up Age	Conclusions
Cascio et al. [51] 2004, Italy	41 exposed/82 controls 5.0 y	Retrospective matched cohort study	yes	Minocycline	2.5 mg/kg twice daily	21 d	Mean age at examination 11.6 y (median interval 7.2 y)	Minocycline could be used to treat infections in pediatric patients when indicated
Volovitz et al. [52] 2007, Israel	31 exposed + 30 controls/4.1 y	Retrospective controlled follow-up study	yes	Doxycycline	4 mg/kg bid on day 1, then 2 mg/kg/day for 9 days	10 d	Mean age at examination 10.4 y (6.3 years since first treatment)	Treatment with doxycycline in c aged 2–8 y is not associated with tooth staining.
Todd et al. [53] 2015, USA	58 c/4.5 y	Retrospective cohort study	Yes	Doxycycline	Mean dose 2.3 mg/kg/dose, typically twice daily	Mean of 7.3 d	Mean age at examination 9.8 y	Short-term doxycycline exposure before 8 years of age was not associated with visible dental staining, enamel hypoplasia or tooth shade differences
Poyhonen et al. [54] 2017, Finland	38 c/4.7 y	Retrospective observational follow-up study	no	Doxycycline	10 mg/kg/day for first 2–3 days, then 5 mg/kg/day	Mean 12.5 days (range 2–28)	Mean age at examination 14.2 y (mean follow-up 9.6 y)	Doxycycline treatment of small children does not seem to induce permanent tooth staining.
Zhu et al. [55] 2021, China	12 children/median exposure age 5.2 y	Pilot retrospective follow-up study	no	Tigecycline	Mean daily dose 2.3 ± 0.6 mg/kg/day	Median 12.5 d (IQR 8.0–19.3)	Median age at examination 9.1 y (4.5 years after exposure)	Tigecycline may cause yellow staining of permanent teeth, although further follow-up is needed
Brown et al. [56] 2023, USA	32 children (18 evaluated for dental staining)/mean age 5.1	Retrospective case series with telephone follow-up	no	Doxycycline	4.5 mg/kg/day divided twice daily.	Mean 12.5 d	NR	Doxycycline is generally well tolerated and an effective treatment for Lyme disease in young children. Prospective observational studies with long-term assessment are needed.

**Table 4:** Dental staining outcomes in children exposed to tetracycline derivatives during tooth development.

Authors, Year, Country	Antibiotic	Exposed (n)	Controls (n)	Dental Staining Exposed (%)	Dental Staining Controls (%)	Statistical Comparison
Cascio et al. [51] 2004 (Italy)	Minocycline	41	82	2/41 (4.9)	2/82 (2.4)	NS
Volovitz et al. [52] 2007 (Israel)	Doxycycline	31	30	0/31 (0)	0/30 (0)	NS
Todd et al. [53] 2015 (USA)	Doxycycline	58	213	0/58 (0)	0/213 (0)	NS
Pöyhönen et al. [54] 2017 (Finland)	Doxycycline	38	No control group	0/38 (0)	NR	Not applicable
Zhu et al. [55] 2021 (China)	Tigecycline	12	No control group	2/12 (16.7)	NR	Not applicable
Brown et al. [56] 2023 (USA)	Doxycycline	18 evaluated	No control group	2/18 (11.1)	NR	Not applicable

Dental staining was assessed clinically by trained dentists in all studies except Brown et al., where outcomes were parent-reported. Brown et al. [56]: 18 of 32 enrolled children completed dental follow-up. Cascio et al. [51]: dental discoloration (2/41) was distinguished from overall enamel defects (14/41).

**Table 5:** Methodological quality assessment of the non-comparative observational studies using the JBI Critical Appraisal Checklist for Analytical Cross-Sectional Studies.

Study	Were the Criteria for Inclusion in the Sample Clearly Defined?	Were the Study Subjects and the Setting Described in Detail?	Was the Exposure Measured in a Valid and Reliable Way?	Were Objective, Standard Criteria Used for Measurement of the Condition?	Were Confounding Factors Identified?	Were Strategies to Deal with Confounding Factors Stated?	Were the Outcomes Measured in a Valid and Reliable Way?	Was Appropriate Statistical Analysis Used?	Score
Zhu et al. [55]	Yes	Yes	Yes	Yes	No	No	Yes	Yes	6/8
Brown et al. [56]	Yes	Yes	Yes	Yes	Yes	No	Yes	Yes	7/8

**Table 6:** Methodological quality assessment of the retrospective cohort studies using the Newcastle–Ottawa Scale (NOS).

Study	Representativeness of Exposed Cohort	Selection of Non-Exposed Cohort	Ascertainment of Exposure	Outcome Not Present at Baseline	Comparability of Cohorts	Outcome Assessment	Adequate Follow-Up Duration	Adequacy of Follow-Up	Total Score
Cascio et al. [51]	Yes	Yes	Yes	Yes	Yes	Yes	Yes	Yes	8/8
Volovitz et al. [52]	Yes	Yes	Yes	Yes	Yes	Yes	Yes	No	7/8
Todd et al. [53]	Yes	Yes	Yes	Yes	Yes	Yes	Yes	Yes	8/8
Pöyhönen et al. [54]	Yes	No	Yes	Yes	No	Yes	Yes	Yes	6/8

**Table 7:** Summary of Findings (GRADE).

Outcome	No. of Studies (Design)	Risk of Bias	Inconsistency	Indirectness	Imprecision	Other Considerations	Overall Certainty of Evidence
Permanent dental staining after doxycycline/minocycline exposure in children < 8 years	5 observational studies (4 retrospective cohorts, 1 retrospective case series)	Not serious	Not serious	Not serious	Serious	None	⊕⊕◯◯ LOW
Permanent dental staining after tigecycline exposure in children < 8 years	1 observational study (retrospective case series)	Serious	Not serious	Not serious	Very serious	None	⊕◯◯◯ VERY LOW

⊕⊕◯◯: low certainty of evidence; ⊕◯◯◯: very low certainty of evidence;

## Data Availability

No new data were created or analyzed in this study. Data sharing is not applicable to this article.

## Supplementary Materials

The following supporting information can be downloaded at: https://www.mdpi.com/article/10.3390/children13081029/s1, File S1: PRISMA 2020 Checklist [19].

## References

[1] Nelson M.L., Levy S.B. (2011). The history of the tetracyclines. Ann. N. Y. Acad. Sci..

[2] Shwachman H., Schuster A. (1956). The tetracyclines: Applied pharmacology. Pediatr. Clin. N. Am..

[3] Grossman E.R., Walchek A., Freedman H. (1971). Tetracyclines and permanent teeth: The relation between dose and tooth color. Pediatrics.

[4] Forti G., Benincori C. (1969). Doxycycline and the teeth. Lancet.

[5] Wormser G.P., Wormser R.P., Strle F., Myers R., Cunha B.A. (2019). How safe is doxycycline for young children or for pregnant or breastfeeding women?. Diagn. Microbiol. Infect. Dis..

[6] Heaton P.C., Fenwick S.R., Brewer D.E. (2007). Association between tetracycline or doxycycline and hepatotoxicity: A population-based case–control study. J. Clin. Pharm. Ther..

[7] Cohlan S.Q., Bevelander G., Tiamsic T. (1963). Growth inhibition of prematures receiving tetracycline. Am. J. Dis. Child..

[8] Guggenheimer J. (1984). Tetracyclines and the human dentition. Compend. Contin. Educ. Dent..

[9] Esposito S., Blasi F., Arosio C., Fioravanti L., Fagetti L., Droghetti R., Tarsia P., Allegra L., Principi N. (2000). Importance of acute Mycoplasma pneumoniae and Chlamydia pneumoniae Doxycycline Treatment in Young Children in children with wheezing. Eur. Respir. J..

[10] Conchie J.M., Munroe J.D., Anderson D.O. (1970). The incidence of staining of permanent teeth by the tetracyclines. Can. Med. Assoc. J..

[11] Ma K., Lu M., Li H., Yuan X., Zhang Y., Ni Q., Li Y., Dong X., Guo J. (2025). Incidence and influencing factors of tooth discoloration in children using doxycycline: A meta-analysis. Front. Pediatr..

[12] Boast A., Curtis N., Gwee A. (2016). QUESTION 1: Teething issues: Can doxycycline be safely used in young children?. Arch. Dis. Child..

[13] Nahum G.G., Kennedy D.L. (2006). Antibiotic use in pregnancy and lactation. What is and is not known about teratogenic and toxic risks. Obstet. Gynecol..

[14] McIntosh H.A., Storey E. (1970). Tetracycline-induced tooth changes, part 4. Discoloration and hipoplasia induced by tetracycline analogues. Med. J. Aust..

[15] Crossa R., Linga C., Nicholas P.J., McGread R., Paris D.H. (2016). Revisiting doxycycline in pregnancy and early childhood—Time to rebuild its reputation?. Expert. Opin. Drug Saf..

[16] Muanda F.T., Sheehy O., Berard A. (2017). Use of antibiotics during pregnancy and risk of spontaneous abortion. Can. Med. Assoc. J..

[17] Muanda F.T., Sheehy O., Berard A. (2017). Use of antibiotics during pregnancy and the risk of major congenital malformations: A population based cohort study. Br. J. Clin. Pharmacol..

[18] Committee on Infectious Diseases, American Academy of Pediatrics (2018). Red Book.

[19] Page M.J., McKenzie J.E., Bossuyt P.M., Boutron I., Hoffmann T.C., Mulrow C.D., Shamseer L., Tetzlaff J.M., Akl E.A., Brennan S.E. (2021). The PRISMA 2020 statement: An updated guideline for reporting systematic reviews. BMJ.

[20] Hoogendam A., de Vries Robbe P.F., Overbeke A.J. (2012). Comparing patient characteristics, type of intervention, control, and outcome (PICO) queries with unguided searching: A randomized controlled crossover trial. J. Med. Libr. Assoc..

[21] AlSaeed T., Nosrat A., Melo M.A., Wang P., Romberg E., Xu H., Fouad A.F. (2018). Antibacterial Efficacy and Discoloration Potential of Endodontic Topical Antibiotics. J. Endod..

[22] Venkataraman M., Singhal S., Tikku A.P., Chandra A. (2019). Comparative analysis of tooth discoloration induced by conventional and modified triple antibiotic pastes used in regenerative endodontics. Indian J. Dent. Res..

[23] Küçükekenci F., Çakici F., Küçükekenci A.S. (2019). Spectrophotometric analysis of discoloration and internal bleaching after use of different antibiotic pastes. Clin. Oral. Investig..

[24] Porter M.L., Münchow E.A., Albuquerque M.T., Spolnik K.J., Hara A.T., Bottino M.C. (2016). Effects of Novel 3-dimensional Antibiotic-containing Electrospun Scaffolds on Dentin Discoloration. J. Endod..

[25] Altun N.B., Turkyilmaz A. (2024). Potential Crown Discoloration Induced by the Combination of Various Intracanal Medicaments and Scaffolds Applied in Regenerative Endodontic Therapy. Niger. J. Clin. Pract..

[26] Kirchhoff A.L., Raldi D.P., Salles A.C., Cunha R.S., Mello I. (2015). Tooth discolouration and internal bleaching after the use of triple antibiotic paste. Int. Endod. J..

[27] Nagata J.Y., Soares A.J., Souza-Filho F.J., Zaia A.A., Ferraz C.C., Almeida J.F., Gomes B.P. (2014). Microbial evaluation of traumatized teeth treated with triple antibiotic paste or calcium hydroxide with 2% chlorhexidine gel in pulp revascularization. J. Endod..

[28] Tay F.R., Mazzoni A., Pashley D.H., Day T.E., Ngoh E.C., Breschi L. (2006). Potential iatrogenic tetracycline staining of endodontically treated teeth via NaOCl/MTAD irrigation: A preliminary report. J. Endod..

[29] Akcay M., Arslan H., Yasa B., Kavrık F., Yasa E. (2014). Spectrophotometric analysis of crown discoloration induced by various antibiotic pastes used in revascularization. J. Endod..

[30] Kim J.H., Kim Y., Shin S.J., Park J.W., Jung I.Y. (2010). Tooth discoloration of immature permanent incisor associated with triple antibiotic therapy: A case report. J. Endod..

[31] Mandras N., Roana J., Allizond V., Pasqualini D., Crosasso P., Burlando M., Banche G., Denisova T., Berutti E., Cuffini A.M. (2013). Antibacterial efficacy and drug-induced tooth discolouration of antibiotic combinations for endodontic regenerative procedures. Int. J. Immunopathol. Pharmacol..

[32] Bremell D., Trollfors B. (2017). Doxycycline can be given to children without risk of staining of teeth. Lakartidningen.

[33] Li Z.Y., Cheng X.R., Wang Y.N. (2004). A preliminary study on the color effect of IPS Empress all-ceramic veneers. Zhonghua Kou Qiang Yi Xue Za Zhi.

[34] Chen J., Shi C., Wang M., Zhao S., Wang H. (2003). Clinical evaluation of 546 tetracycline-stained teeth treated with Cerinate laminate veneers. Zhonghua Kou Qiang Yi Xue Za Zhi.

[35] Bennett Z.Y., Walsh L.J. (2015). Effect of Photo-Fenton Bleaching on Tetracycline-stained Dentin in vitro. J. Contemp. Dent. Pract..

[36] Newman J., Youn T., Suk M., Dong C., Lu J., Sample M., Yang I., Chan D.C. (2025). Assessments of a novel bleaching agent containing sodium dithionite: A laboratory study. Am. J. Dent..

[37] Chan D.C., Rozier G.S., Steen A., Browning W.D., Mozaffari M.S. (2006). Standardized method to produce tetracycline-stained human molar teeth in vitro. Quintessence Int..

[38] Fukuta Y., Takeda Y., Fukuta Y., Totsuka M., Yoshida Y., Hayashi S., Niitsu J., Yamamoto H. (2001). An unusual staining of the tooth roots: A case report with histological and micro-analytical studies. J. Oral Sci..

[39] Metz L.M., Li D.K., Traboulsee A.L., Duquette P., Eliasziw M., Cerchiaro G., Greenfield J., Riddehough A., Yeung M., Kremenchutzky M. (2017). Trial of Minocycline in a Clinically Isolated Syndrome of Multiple Sclerosis. N. Engl. J. Med..

[40] Tapananon A., Kamolroongwarakul P., Buranaprasertsuk P. (2025). Minimally Invasive Multidisciplinary Management of Tetracycline-stained and Congenitally Missing Teeth. J. Contemp. Dent. Pract..

[41] Leonard R.H., Van Haywood B., Caplan D.J., Tart N.D. (2003). Nightguard vital bleaching of tetracycline-stained teeth: 90 months post treatment. J. Esthet. Restor. Dent..

[42] Gimeno I., Riutord P., Tauler P., Tur J.A., Pons A. (2008). The whitening effect of enzymatic bleaching on tetracycline. J. Dent..

[43] Deliperi S., Congiu M.D., Bardwell D.N. (2006). Integration of composite and ceramic restorations in tetracycline-bleached teeth: A case report. J. Esthet. Restor. Dent..

[44] Chen J.H., Shi C.X., Wang M., Zhao S.J., Wang H. (2005). Clinical evaluation of 546 tetracycline-stained teeth treated with porcelain laminate veneers. J. Dent..

[45] Matis B.A., Wang Y., Jiang T., Eckert G.J. (2002). Extended at-home bleaching of tetracycline-stained teeth with different concentrations of carbamide peroxide. Quintessence Int..

[46] Ayaslioglu E., Erkek E., Oba A.A., Cebecioğlu E. (2005). Doxycycline-induced staining of permanent adult dentition. Aust. Dent. J..

[47] Joshi G., Dhingra D., Pandav S.S., Kaushik S. (2020). Doxycycline-induced staining of teeth and malar rash in a child. J. Postgrad. Med..

[48] Moola S., Munn Z., Tufanaru C., Aromataris E., Sears K., Sfetcu R., Currie M., Lisy K., Qureshi R., Mattis P., Aromataris E., Lockwood C., Porritt K., Pilla B., Jordan Z. (2024). Systematic reviews of etiology and risk. Joanna Briggs Institute Reviewer’s Manual.

[49] Wells G.A., Shea B., O’Connell D., Peterson J., Welch V., Losos M., Tugwell P. (2000). The Newcastle–Ottawa Scale (NOS) for Assessing the Quality of Nonrandomised Studies in Meta-Analyses.

[50] Guyatt G.H., Oxman A.D., Vist G.E., Kunz R., Falck-Ytter Y., Alonso-Coello P., Schünemann H.J. (2008). GRADE: An emerging consensus on rating quality of evidence and strength of recommendations. BMJ.

[51] Cascio A., Di Liberto C., D’Angelo M., Iaria C., Scarlata F., Titone L., Campisi G. (2004). No Findings of Dental Defects in Children Treated with Minocycline. Antimicrob. Agents Chemother..

[52] Volovitz B., Shkap R., Amir J., Calderon S., Varsano I., Nussinovitch M. (2007). Absence of Tooth Staining with Doxycycline Treatment in Young Children. Clin. Pediatr..

[53] Todd S.R., Dahlgren F.S., Traeger M.S., Beltran-Aguilar E.D., Marianos D.W., Hamilton C., McQuiston J.H., Regan J. (2015). No Visible Dental Staining in Children Treated with Doxycycline for Suspected Rocky Mountain Spotted Fever. J. Pediatr..

[54] Poyhonen H., Nurmi M., Peltola V., Alaluusua S., Ruuskanen O., Lahdesmaki1 T. (2017). Dental staining after doxycycline use in children. J. Antimicrob. Chemother..

[55] Zhu Z., Yu Q., Qi G., Yang J., Ni Y., Ruan W., Fang L. (2021). Tigecycline-Induced Tooth Discoloration in Children Younger than Eight Years. Antimicrob. Agents Chemother..

[56] Brown K., Corin S., Handel A.S. (2023). Doxycycline for the Treatment of Lyme Disease in Young Children. Pediatr. Infect. Dis. J..

[57] Shwachman H., Feket E., Kulczycki L., Foley G.E. (1958). The effect of long-term antibiotic therapy in a patient wit cystic fibrosis of the pancreas. Antibiot. Annu..

[58] Porter P.J., Sweeney E.A., Golan H., Kass E.H. (1965). Controlled study of the effect of prenatal tetracycline on primary dentition. Antimicrob. Agents Chemother..

[59] Department of Health Education and Welfare (1978). Tetracycline pediatric drops to be withdrawn from market. FDA Bull..

[60] Wallman I.S., Hilton H.B. (1962). Teeth pigmented by tetracycline. Lancet.

[61] Swallow J.N., De Haller J., Young W.F. (1967). Side-effects to antibiotics in cystic fibrosis: Dental changes in relation to antibiotic administration. Arch. Dis. Child..

[62] Rebich T., Kumar J., Brustman B. (1983). The St. Regis environmental health issue: Assessment of dental defects. J. Am. Dental Assoc..

[63] Lochary M.E., Lockhart P.B., Williams W.T. (1998). Doxycycline and staining of permanent teeth. Pediatr. Infect. Dis. J..

[64] Cale D.F., McCarthy M.W. (1997). Treatment of Rocky Mountain spotted fever in children. Ann. Pharmacother..

[65] Lantos P.M., Rumbaugh J., Bockenstedt L.K., Falck-Ytter Y.T., Aguero-Rosenfeld M.E., Auwaerter P.G., Baldwin K., Bannuru R.R., Belani K.K., Bowie W.R. (2021). Clinical practice guidelines by the Infectious Diseases Society of America (IDSA), American Academy of Neurology (AAN), and American College of Rheumatology (ACR): 2020 guidelines for the prevention, diagnosis and treatment of Lyme disease. Clin. Infect. Dis..

[66] Cascio A., Scarlata F., Giordano S., Antinori S., Colomba C., Titone L. (2003). Treatment of human brucellosis with rifampin plus minocycline. J. Chemother..

[67] Eady E.A., Cove J.H., Holland K.T., Cunliffe W.J. (1990). Superior antibacterial action and reduced incidence of bacterial resistance in minocycline compared to tetracycline-treated acne patients. Br. J. Dermatol..

[68] Jawetz E., Katzung B. (1989). Chloramphenicol and tetracycline. Basic and Clinical Pharmacology.

[69] Rang H.P., Dale M.M., Ritter J.M. (1995). Pharmacology.

[70] Saivin S., Houin G. (1988). Clinical pharmacokinetics of doxycycline and minocycline. Clin. Pharmacokinet..

[71] Stein G.E., Craig W.A. (2006). Tigecycline: A critical analysis. Clin. Infect. Dis..

[72] Pfaller M.A., Huband M.D., Streit J.M., Flamm R.K., Sader H.S. (2018). Surveillance of tigecycline activity tested against clinical isolates from a global (North America, Europe, Latin America and Asia-Pacific) collection (2016). Int. J. Antimicrob. Agents.

[73] Lin S., Zhang C., Ye S. (2018). Preliminary experience of tigecycline treatment for infection in children with hematologic malignancies. Int. J. Clin. Pharm..

[74] Ye S., Zhang C., Lin S. (2018). Preliminary experience with tigecycline treatment for severe infection in children. Eur. J. Pediatr..

[75] Zeng J., Zhang L., Gao M., Wu J., Wu H., Chen J., Chen X., Tang W. (2017). Tigecycline treatment in an infant with extensively drug-resistant Acinetobacter baumannii bacteremia. Int. J. Infect. Dis..

[76] Zhu Z.Y., Yang J.F., Ni Y.H., Ye W.F., Wang J., Wu M.L. (2016). Retrospective analysis of tigecycline shows that it may be an option for children with severe infections. Acta Paediatr..

[77] Lin S., Liang L., Zhang C., Ye S. (2020). Preliminary experience of tigecycline treatment in critically ill children with ventilator-associated pneumonia. J. Int. Med. Res..

[78] Peng C., Wang X., Zhang J., Jiang Y., Hou X. (2018). Tigecycline application in a 3-month-old infant with multiple drug resistant Klebsiella pneumonia: A case report. Gut Pathog..

[79] Song Y., Hu L., Shu Q., Ye J., Liang J., Chen X., Tan L. (2018). Tigecycline salvage therapy for critically ill children with multidrug-resistant/extensively drug-resistant infections after surgery. Int. J. Infect. Dis..

[80] Rajan A.S., Gopal M., Periyathambi M., Kuttiatt V.S. (2025). Dental safety of short-term doxycycline use in children under 8 years: A systematic review and meta-analysis. Front. Pharmacol..

